# Blockage of NLRP3 inflammasome activation ameliorates acute inflammatory injury and long-term cognitive impairment induced by necrotizing enterocolitis in mice

**DOI:** 10.1186/s12974-021-02111-4

**Published:** 2021-03-06

**Authors:** Fangxinxing Zhu, Lingyu Wang, Zizhen Gong, Yanyan Wang, Yanhong Gao, Wei Cai, Jin Wu

**Affiliations:** 1 Department of Pediatric Surgery, Xinhua hospital, School of Medicine, Shanghai Jiaotong University, Shanghai, China; 2 Shanghai Institute for Pediatric Research, School of Medicine, Shanghai Jiaotong University, Shanghai, China; 3 Shanghai Key Laboratory of Pediatric Gastroenterology and Nutrition, Shanghai, China; 4 Department of Geriatrics, Xinhua Hospital, School of Medicine, Shanghai Jiaotong University, Shanghai, China

**Keywords:** Neonatal necrotizing enterocolitis, NLRP3 inflammasome, IL-1β, Intestinal injury, Brain damage, Cognitive impairment, MCC950

## Abstract

**Background:**

Necrotizing enterocolitis (NEC) is an inflammatory gastrointestinal disease in premature neonates with high mortality and morbidity, while the underlining mechanism of intestinal injury and profound neurological dysfunction remains unclear. Here, we aimed to investigate the involvement of NLPR3 inflammasome activation in NEC-related enterocolitis and neuroinflammation, especially long-term cognitive impairment, meanwhile, explore the protective effect of NLRP3 inhibitor MCC950 on NEC in mice.

**Methods:**

NLRP3 inflammasome activation in the intestine and brain was assessed in the NEC mouse model, and NLRP3 inhibitor MCC950 was administrated during the development of NEC. Survival rate, histopathological injury of the intestine and brain, and expression of mature IL-1β and other pro-inflammatory cytokines were analyzed. Long-term cognitive impairment was evaluated by behavioral test.

**Results:**

The expression of NLRP3 and mature IL-1β in the intestine and brain was greatly upregulated in NEC mice compared to the controls. MCC950 treatment efficiently improved NEC survival rate, reduced intestinal and brain inflammation, and ameliorated the severity of pathological damage in both organs. Additionally, in vivo blockage of NLRP3 inflammasome with MCC950 in early life of NEC pups potently protected against NEC-associated long-term cognitive impairment.

**Conclusions:**

Our findings suggest that NLRP3 inflammasome activation participates in NEC-induced intestinal and brain injury, and early intervention with NLRP3 inhibitor may provide beneficial therapeutic effect on NEC infants.

**Supplementary Information:**

The online version contains supplementary material available at 10.1186/s12974-021-02111-4.

## Background

Neonatal necrotizing enterocolitis (NEC) is a lethal gastrointestinal disease in preterm neonates with high morbidity, and about 3–15% of preterm infants will develop NEC [1]. Due to the deficiency of effective treatments and increasing number of premature infants, the overall NEC mortality has sustained at high level ranging from 20 to 30%, and NEC patients that have undergone surgery exhibit even higher mortality at around 50% [2]. Moreover, multiple severe early and long-term complications, including enterobrosis, septic shock, short bowel syndrome, and profound neurological impairments, have been widely reported in NEC survivors [3], therefore necessitating novel therapeutic approaches to be explored.

Although the precise etiology of NEC remains poorly understood, it is generally believed that NEC occurrence is associated with multiple factors, such as prematurity, intestinal mucosal ischemia and hypoxia, abnormal intestinal flora colonization, and non-breast feeding [4, 5]. The exaggerated inflammatory response induced by combined effect of risk factors mentioned above plays an important role in the pathogenesis of NEC, contributing to intestinal ischemic necrosis, enterobrosis, and systemic symptoms [6]. Among diverse pro-inflammatory cytokines, the involvement of interleukin-1β (IL-1β) in the development of NEC has been commonly described. Clinical studies observed that the intestinal mRNA levels of IL-1β significantly increased in NEC patients compared with normal controls [7]. More importantly, serum IL-1β levels appear to correlate with disease activity, as evidenced by the fact that compared to the serum IL-1β levels of NEC patients in clinical stages I and II, those of patients in stage III have increased further. Moreover, IL-1β could trigger the release of other potent inflammatory cytokines, including interleukin-6 (IL-6) and tumor necrosis factor-α (TNF-α), thus comprising the vicious cycle to aggravate inflammatory damage in the intestine [8, 9]. Numerous studies have proved that the production of IL-1β relies on nucleotide-binding and oligomerization domain (NOD)-like receptor protein 3 (NLRP3) inflammasome, the most fully characterized inflammasome. As an important part of innate immune system, NLRP3 inflammasome mediates the cleavage of caspase-1 and consequently catalyzes pro-IL-1β to form mature IL-1β upon stimulation [10]. Accumulating evidence has demonstrated that excessive NLRP3 inflammasome activation and IL-1β production participate in the development of various inflammatory diseases and thus could possibly serve as candidate therapeutic targets [11]. Of note, a recent study disclosed that NLRP3 expression level was upregulated in the intestine of NEC rat model [12], suggesting the involvement of NLRP3 inflammasome activation in intestinal inflammatory response of NEC; however, whether it is also implicated in NEC-related brain injury and long-term cognitive decline remains uncovered. Moreover, the therapeutic effect of NLRP3 inhibition on NEC also needs to be evaluated.

In the present study, by using a NEC mouse model, we proved the contribution of NLRP3 inflammasome to NEC and NEC-induced brain injuries. We further explored whether a selective NLRP3 inhibitor MCC950 could alleviate NEC-related intestinal and neuroinflammation, especially long-term cognitive impairments, therefore providing a new potential therapeutic strategy for NEC.

## Materials and methods

### Animals

All the animal experiments and procedures were in accordance with the Guide for the Care and Use of Medical Laboratory Animals issued by the Ministry of Health of China and approved by the Ethics Committee of Xinhua Hospital Affiliated to Shanghai Jiaotong University School of Medicine. Seven-day-old C57BL/6 mice were housed in Experimental Animal Center of Xinhua Hospital Affiliated to Shanghai JiaoTong University School of Medicine (Shanghai, China). All the mice were maintained on a 12-h/12-h light/dark cycle in a humidity-controlled specific pathogen-free facility.

### NEC induction and drug treatment

Experimental NEC mouse model was established as described previously with a little modification [13]. In brief, 7-day-old mice were fed by gavage with hyperosmolar formula [Similac Advance infant formula (Abbott Nutrition) and Esbilac canine milk replacer (PetAg) at a ratio of 2:1] every 3 h, meanwhile, exposed to transient hypoxia (5% O_2_, 10 min) and hypothermia twice daily for 4 days. In some cases, pups in the NEC group received an intraperitoneal injection of MCC950 (10 mg/kg, Selleck) once a day for 4 consecutive days (NEC+MCC950 group). In addition, age-matched and unstressed breastfed pups served as control group. Body weight was recorded daily throughout the course of experiment. On day 5, mice were sacrificed, and the brain as well as intestinal tissues was harvested for further analysis.

For the cognitive evaluation study, some pups in NEC and NEC+MCC950 groups were kept breast-feeding after NEC induction until weaned and then subjected to Morris water maze test at 28-day-old. Age-matched breastfed mice without treatment were regarded as control.

### Gut histology

The paraffin sections of intestinal tissue were stained with hematoxylin and eosin, and an established scoring criteria was applied to assess the histological injury in a double-blinded manner [14] (control group, *n*=5; NEC group, *n*=6; NEC+MCC950 group, *n*=9). The detailed histological grade was as follows: (1) normal, intact morphology; (2) mild, separation of the villus core without other abnormalities; (3) moderate, villus core separation, submucosal edema, and epithelial sloughing; (4) severe, denudation of epithelium with loss of villi, full thickness necrosis, or perforation. Specimens with moderate or severe histological abnormalities were considered consistent with NEC.

### Brain gross anatomy and histology

The whole brain was removed and weighed. All the brains were then fixed in paraformaldehyde (4%) and embedded in paraffin. Sagittal-oriented sections (5 μm) were stained with hematoxylin and eosin. Evaluation of hippocampal neuronal damage was performed in a blinded manner as described previously [15] (control group, *n*=5; NEC group, *n*=7; NEC+MCC950 group, *n*=5). Normal neurons are arranged uniformly and have abundant cytoplasm with large round nuclei, while damaged neurons are disorderly arranged and exhibit shrunken cell bodies with pyknotic nuclei. Briefly, no damage to any hippocampal subregion was scored as 0, scattered damaged neurons in the CA1 subregion was scored as 1, moderate numbers of damaged neurons in the CA1 subregion (< 50%) was scored as 2, severe neuron damage in the CA1 subregion (> 50%) was scored as 3, and extensive neuron damage in all hippocampal regions was scored as 4.

### Immunohistochemistry

Paraffin sections from all groups (*n*=4–6 pups/group) were deparaffinized before analyzing for NLRP3, IL-1β, and ZO-1. Briefly, murine intestinal or brain tissue sections were soaked in xylene and serially passed through decreasing concentrations of ethanol, followed by antigen retrieval. Then, slides were incubated overnight at 4 °C with the following primary antibodies respectively against NLRP3 (Abcam, Cambridge), IL-1β (Cell Signaling). After incubating with the appropriate secondary antibody for 60 min, slides were stained with 3,3′-diaminobenzidine, and nuclei were stained using hematoxylin.

### Western blot

Brain or intestinal tissue proteins were extracted using a tissue lysis buffer (Sigma) supplemented with proteinase and phosphatase inhibitors (Thermo Fisher Scientific). Protein concentrations were determined by Pierce BCA Protein Assay Kit (Thermo Fisher Scientific). Thirty-five micrograms of total proteins was resolved by SDS-PAGE, transferred to 0.2 μm PVDF membranes, and probed using anti-NLRP3 (Abcam, Cambridge), anti-caspase-1 (Chemicon), anti-IL-1β (Cell Signaling), anti-myelin basic protein (MBP) (Abcam, Cambridge), anti-NeuN (Abcam, Cambridge), or anti-β-actin antibody (Sigma). Reactive signals were detected using chemiluminescence (Pierce, ECL Western Blotting Substrate, Thermo Fisher Scientific). The density of target bands was quantified by Image J analysis system (Bio-Rad).

### Real-time PCR

Total RNA was isolated from the intestine or brain tissue using a pure yield RNA Mini-prep kit (Promega). cDNAs were synthesized using a Transcriptor First Strand cDNA Synthesis Kit (Roche). The mRNA expression levels of IL-6, TNF-α, NLRP3, NLRP1, NLRC4, and AIM2 were then determined by Real-PCR on a 7300 Real-Time PCR System (Applied Biosystems). The primer sequences were as follows: IL-6, forward, 5′-TAG TCC TTC CTA CCC CAA TTT CC-3′ and reverse, 5′-TTG GTC CTT AGC CAC TCC TTC-3′; TNF-α, forward 5′-GCA TGA TCC GAG ATG TGG AA-3′ and reverse 5′-TGA GAA AGG CTG AGG CAC A -3′; NLRP3, forward, 5′-GAA GAA GAG TGG ATG GGT TTG-3′ and reverse, 5′-CTG CGT GTA GCG ACT GTT GAG-3′; NLRP1, forward, 5′-AGT AAT CTG GAG GGG TTG GAC-3′ and reverse, 5′-GTT GGC AGC CAG GGT ATA TCA-3′; NLRC4, forward, 5′-ATC GTC ATC ACC GTG TGG AG-3′ and reverse, 5′-GCC AGA CTC GCC TTC AAT CA-3′; AIM2, forward, 5′-GTC CTC AAG CTA AGC CTC AGA-3′ and reverse, 5′-CAC CGT GAC AAC AAG TGG AT-3′.

### Morris water maze test

Spatial memory ability was evaluated by Morris water maze (MWM) test [16], which was performed during the light phase between 8 a.m. and 6 p.m. in a sound-attenuated room. Briefly, MWM test consisted of a 5-day acquisition phase and a subsequent 1-day probe test. The experiment was conducted in a circular pool (100 cm in diameter) filled with non-toxic white food colorant-dyed water and maintained at 21–22 °C. The circular pool was equally divided into four quadrants by two mutually perpendicular and bisected virtual axes: target quadrant (T), left quadrant (L, on the left of the target quadrant), right quadrant (R, on the right of the target quadrant), and opposite quadrant (O, on the opposite side of the target quadrant). A round platform (6 cm in diameter) was hidden 1 cm beneath the water surface at a fixed position of the target quadrant. During the 5-day acquisition phase, each mouse was given four swimming trials per day (15-min intertrial interval) to find the hidden platform. The mouse that failed to reach the platform in 60 s was manually guided to the platform and allowed to stay there for 20 s like other mice. Twenty-four hours after the last trial, the platform was removed, and the probe test was performed, which allowed the mice to swim freely for 60 s in the pool. The escape latency to find the platform during the acquisition phase, swim speed, and target crossings (platform-located area) in the probe test was recorded and analyzed by using the Image Automatic Acquisition and Software Analysis System (Xinruan Company, Shanghai).

### Statistics

Results were expressed as the mean ± standard error of the mean. Data analysis was performed by the Graph Pad 5.0 software. For brain weight comparison, parametric test was used. One-way ANOVA followed by Bonferroni’s Multiple Comparison Test and two-way ANOVA followed by Bonferroni post-test were adopted respectively for histological score and behavioral data analysis. Significance was defined as *p* values <0.05.

## Results

### Intestinal injury of murine NEC involves NLRP3 inflammasome activation

**Fig. 1:**
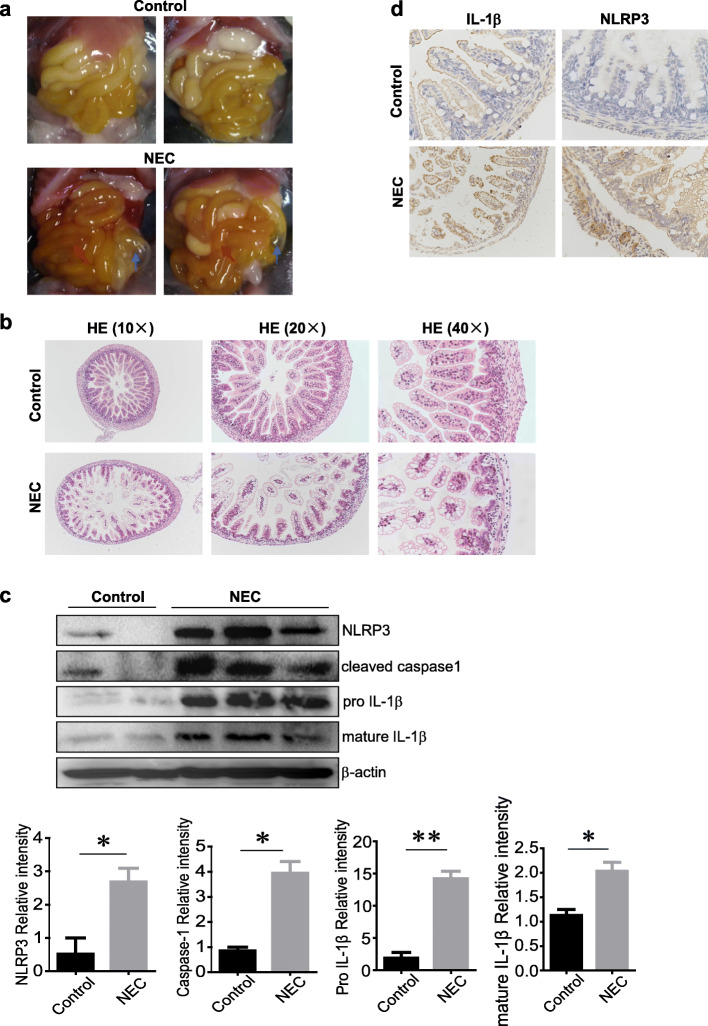
Intestinal injury of murine NEC involves NLRP3 inflammasome activation. **a** Representative gross images of the intestine from the control and NEC group at P11. Blue arrows indicate pneumatosis intestinalis, and red arrow indicates intestine hyperemia. **b** Representative H&E staining of intestinal sections from control and NEC group. **c** Western blot analysis of NLRP3, cleaved caspase-1, pro IL-1β (31kD), and mature IL-1β (17kD) expression in intestinal homogenates. β-actin was regarded as a loading control, and the relative intensity of the bands was quantified (*n*=2–3 mice per group). **d** Representative IL-1β and NLRP3 immunohistochemical staining of intestinal tissues from control and NEC group. Data are presented as mean ± SEM. *p < 0.05, **p < 0.01 vs. control group

To confirm the involvement of NLRP3 inflammasome activation in the development of NEC intestinal injury, we constructed the NEC mouse model with typical disruption of intestinal villus architecture that resembles human NEC, as evidenced by obvious villus disarrangement, villous sloughing with significant villous core separation, and patchy intestinal necrosis (Fig. 1 a and b). NLRP3 inflammasome activation could trigger the caspase-1 cleavage and release of pro-inflammatory cytokines, especially active IL-1β, then participating in diverse inflammatory diseases. Here, we detected the expression of NLRP3, cleaved caspase-1, and pro and mature IL-1β (17kD, active form of IL-1β) in intestinal tissue by western blot, and the results showed that the expression levels of proteins mentioned above all significantly increased in NEC group compared to the control group (Fig. 1c). Consistently, immunohistochemistry staining also exhibited much more expression of IL-1β as well as NLRP3 in NEC group than control (Fig. 1d). Meanwhile, the expression of NLRP1, NLRC4, and AIM2 levels in the intestine of the NEC group were comparable to those in the control group (Supplementary Figure 1); these data indicate that NLRP3 inflammasome at least partially contributes to the intestinal damage of NEC.

### NLRP3 inflammasome activation participates in brain injury of NEC

**Fig. 2:**
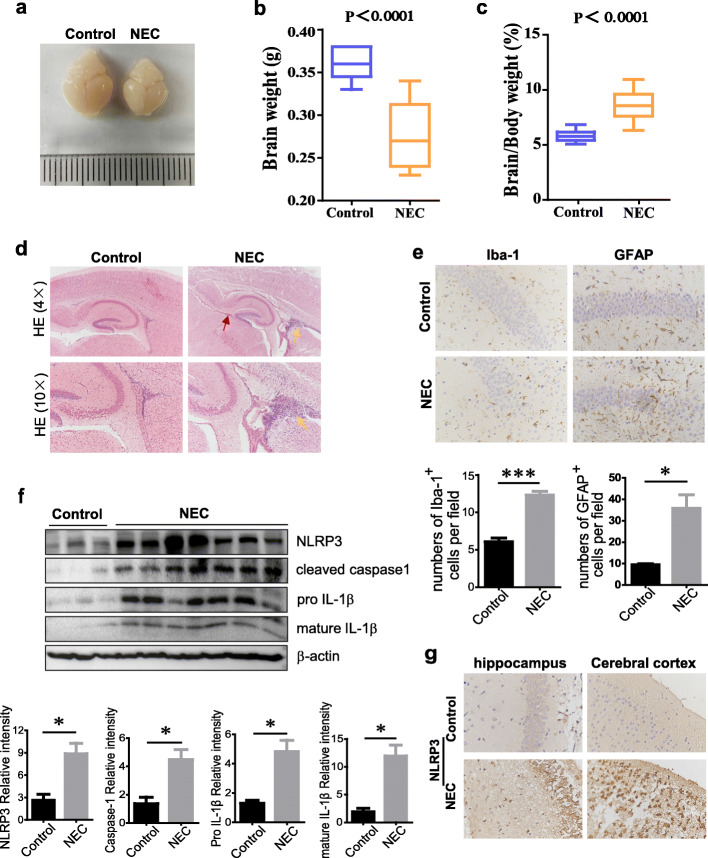
NLRP3 inflammasome activation participates in brain injury of NEC. **a** Representative images of harvested brains from control and NEC mice at P11. Red arrow, hemorrhage; orange arrows, inflammatory cell infiltration. **b** Brain weights and **c** brain/body weight ratios of control and NEC group. Brains in both groups were weighed immediately after harvest. Body weights were measured prior to brain harvest. **d** Representative H&E staining of brain sections from control and NEC group. **e** Representative Iba-1 and GFAP staining in the brain of control and NEC mice. Quantification of IHC staining was exhibited as the number of Iba-1 or GFAP positive cells per field (4–6 fields/mice, *n*=3 mice per group). **f** Western blot analysis of NLRP3, cleaved caspase-1, pro IL-1β, and mature IL-1β protein levels in mouse brain. The relative intensity of the bands was quantified (control, *n* = 3; NEC, *n* = 7). **g** Representative NLRP3 immunohistochemical staining of hippocampus and cerebral cortex regions in control and NEC mice. Data are presented as mean ± SEM. *p < 0.05, ***p < 0.001 vs. control group

In line with the previous reports, the brain of pups in the NEC group was smaller (Fig. 2a) and weighed less (278±12 mg) than that of the control group [(360±6 mg, *p* < 0.0001), Fig. 2b]; meanwhile, the brain/body weight ratio of NEC pups was higher [8.32% (7.90–8.74)] than that of the control [5.74% (5.56–5.92), *p*<0.0001, Fig. 2c]. Furthermore, HE staining on brain sagittal sections showed evident hemorrhage in the adjacent region of hippocampus and inflammatory cell infiltration (Fig. 2d). Emerging evidence has suggested that the existence of neuroinflammation in the brain of individuals suffer from NEC, which may play an important role in acute brain injury and subsequent neurodevelopmental delay. Here, data exhibited more activated microglia and increased number of astrocytes presented in the brain of NEC pups compared with the controls, as evidenced by enhanced Iba-1 and GFAP immunoreactivity (Fig. 2e), confirming the involvement of neuroinflammation in NEC-induced brain injury. Interestingly, our results showed remarkable elevated expression of NLRP3 in both hippocampus and cerebral cortex regions of the brain in NEC pups compared to the breastfed control (Fig. 2g); accordantly, NLRP3, cleaved caspase-1, and pro and mature IL-1β levels of brain tissue were also greatly upregulated in most of the NEC pups (Fig. 2f); these data suggest that NLRP3 inflammasome activation may also participate in the brain inflammatory injury of NEC. To evaluate whether there is a correlation between intestine and brain NLRP3 inflammasome activation, we further compared the IL-1β and IL-18 levels in the intestine and brain of NEC and control pups. Data showed that IL-1β and IL-18 increased significantly in both intestine and brain tissue in NEC pups compared to the breastfed controls. Importantly, a positive correlation between the level of IL-1β in the intestine and in the brain was exhibited (Supplementary Figure 2, *p* = 0.01, *r* = 0.6206). Similarly, we also observed a positive correlation between the level of IL-18 in the intestine and in the brain (*p* = 0.01, *r* = 0.7868). These findings indicate that NLRP3 inflammasome activation in the brain of NEC mice correlates with the inflamed intestine.

### NLRP3 inhibition with MCC950 efficiently ameliorates intestinal injury and improves NEC survival rate in mice

**Fig. 3:**
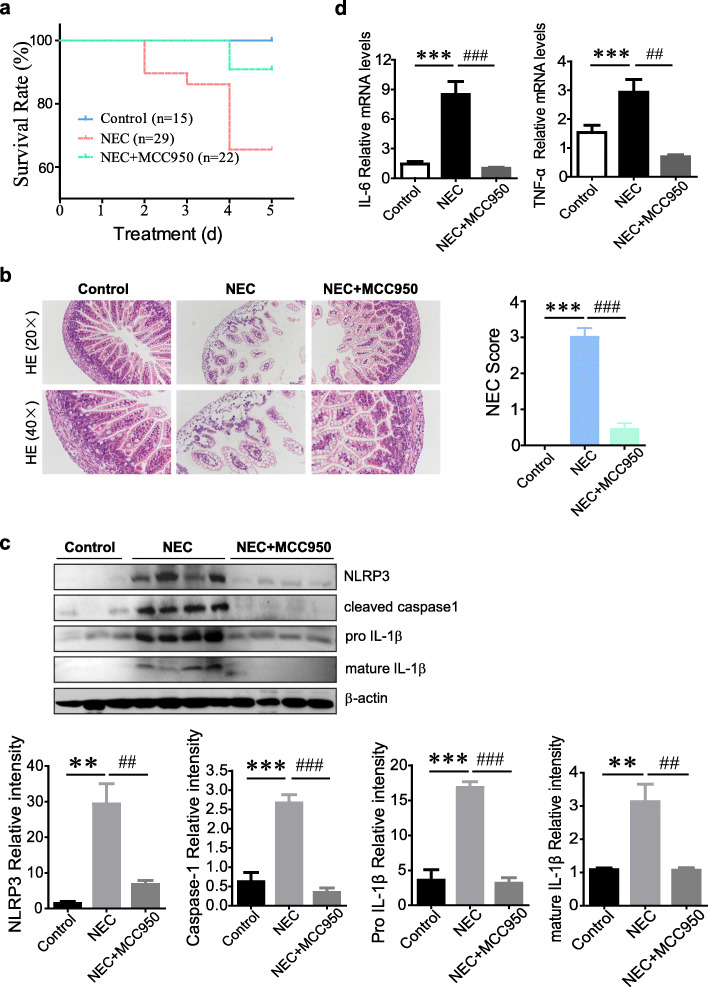
NLRP3 inhibition with MCC950 efficiently ameliorates intestinal injury and improves NEC survival in mice. **a** Survival rate of mice in control, NEC, and NEC+MCC950 groups (*n* = 15–29 mice per group). **b** H&E staining (×20) and histological score of intestinal sections from differently treated mice as described above. **c** Western blot analysis of NLRP3, cleaved caspase-1, pro IL-1β, and mature IL-1β in intestinal homogenates. The relative band intensity was quantified (control, *n* = 3; NEC, *n* = 4; NEC+MCC950, *n* = 4). **d** Relative mRNA expression of IL-6 and TNF-α in intestine (*n* = 5–6 mice per group). Data are presented as mean ± SEM. **p < 0.01, ***p < 0.001 vs. control group; ##*p* < 0.01, ###*p* < 0.001 vs. NEC group

Considering that NEC intestinal injury requires NLRP3 inflammasome activation and IL-1β release, next, we determined to investigate whether MCC950, a specific small molecular inhibitor of NLRP3, could protect against intestinal inflammatory damage in experimental NEC mouse model*.* According to our data, intraperitoneal administration of MCC950 obviously improved the animal survival rate from 65.52% in NEC group to 90.91% in NEC+MCC950 group (*p*<0.05, Fig. 3a), accompanied by significant amelioration of intestinal damage (Fig. 3b), while vehicle-treatment control had no apparent effect on NEC injury (Supplementary Figure 3). As expected, production of NLRP3, cleaved caspase-1, and mature IL-1β in intestinal tissues was almost completely blocked upon MCC950 treatment (Fig. 3c), and importantly, the mRNA levels of IL-6 and TNF-α, another two pivotal inflammatory cytokines commonly increased during NEC, were also reduced potently (Fig. 3d). These results indicate that the selective inhibition of NLRP3 inflammasome could significantly prevent intestinal inflammation and tissue injury of NEC.

### NLRP3 inhibitor MCC950 reverses experimental NEC-induced acute neuroinflammation and brain injury

**Fig. 4:**
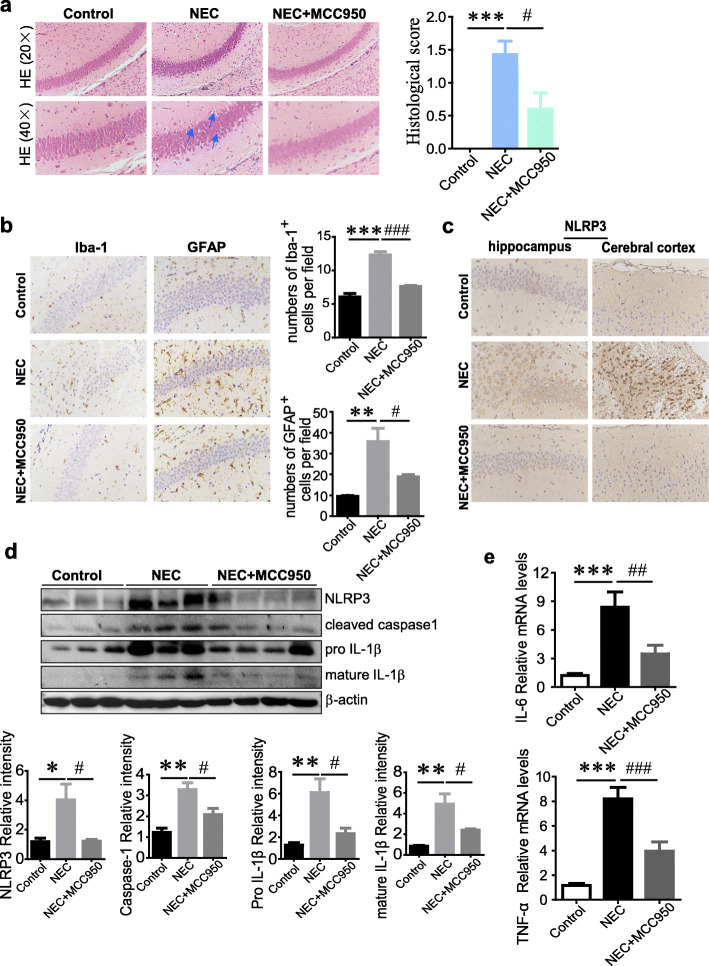
NLRP3 inhibitor MCC950 reverses experimental NEC-induced acute neuroinflammation and brain injury. **a** H&E staining and histological score of hippocampal CA1 regions in control, NEC and NEC+MCC950 mice. Pyramidal cells in NEC mice arranged disorderly with deeply stained cytoplasm and nucleus. Blue arrows indicate nuclear pyknosis. **b** Representative Iba-1 and GFAP staining in brain hippocampus of control, NEC, and NEC+MCC950 mice. The numbers of Iba-1 or GFAP positive cells per field were quantified (4–6 fields/mice, *n* = 3 mice per group). **c** Representative NLRP3 immunohistochemical analysis of hippocampus and cerebral cortex regions. **d** Western blot analysis of NLRP3, cleaved caspase-1, pro IL-1β, and mature IL-1β in mouse brain tissues. The relative band intensity was quantified (control, *n* = 3; NEC, *n* = 3; NEC+MCC950, *n* = 4). **e** Relative mRNA expression of TNF-α and IL-6 in brain (*n* = 5–6 mice per group). Data are presented as mean ± SEM. *p < 0.05, **p < 0.01, ***p < 0.001 vs. control group; #*p* < 0.05, ##*p* < 0.01, ###*p* < 0.001 vs. NEC group

To observe the effect of NLRP3 inhibition by MCC950 on NEC-associated acute injury in the brain, sagittal sections were obtained, and HE staining as well as histological scoring showed that MCC950 treatment could obviously reverse the neuronal damage (Fig. 4a), although it had no significant effect on brain weight and brain/body weight ratio of NEC pups (Supplementary Figure 4). In addition, MCC950 administration largely decreased NEC-associated enhanced Iba-1 and GFAP immunoreactivity in both hippocampus and cerebral cortex regions (Fig. 4b and Supplementary Figure 5). Meanwhile, MCC950 successfully inhibited the NLRP3 inflammasome activation by reducing NLRP3-positive cells in the brain (Fig. 4c) and suppressing NLRP3, cleaved caspase-1, and mature IL-1β generation (Fig. 4d) triggered by NEC. Consistent with the effects of MCC950 on the intestine, the increased expression of IL-6 and TNF-α in response to NEC were also dramatically blunted by MCC950 treatment (Fig. 4e).

### MCC950 administration in early life induces long-term protection from NEC-associated cognitive impairments in mice

**Fig. 5:**
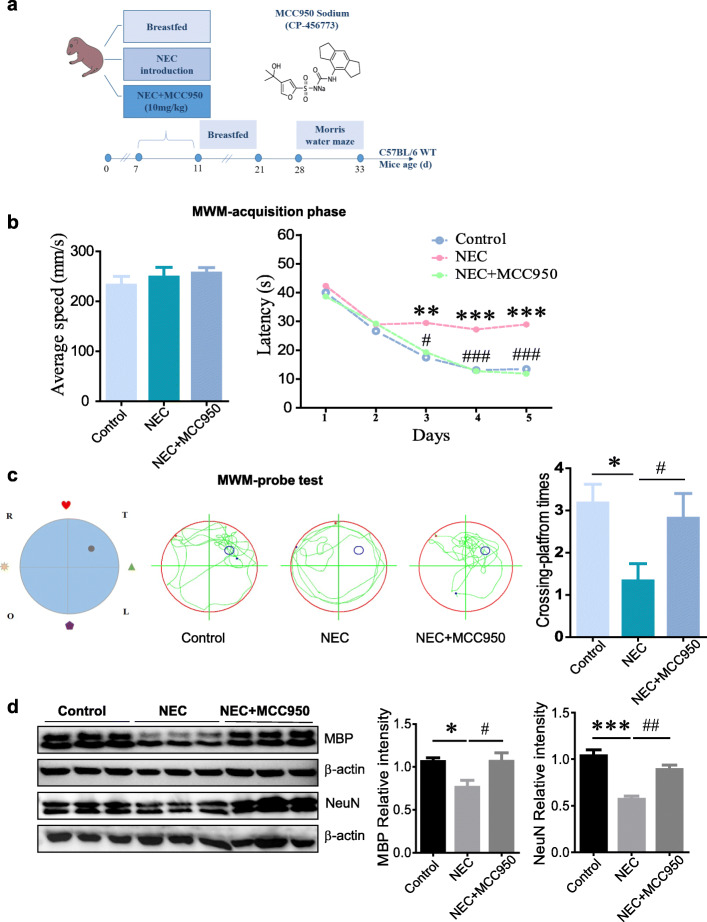
MCC950 administration in early life induces long-term protection from NEC-associated cognitive impairments in mice. **a** Schematic diagram of the experimental design. **b** Average swim speed at the first day and escape latency during the MWM spatial training phase. **c** Representative swim paths and crossing-platform times in the MWM probe test (*n* = 10–11 mice per group). **d** Western blot analysis of MBP and NeuN levels in brain homogenates. The relative intensity of the bands was quantified (*n* = 3 mice per group). Values are presented as mean ± SEM. *p < 0.05, **p < 0.01, ***p < 0.001 vs. control group; #*p* < 0.05, ##*p* < 0.01, ###*p* < 0.001 vs. NEC group

To further evaluate the long-term neuroprotective effects of NLRP3 inhibitor MCC950 on NEC-induced cognitive impairments, Morris water maze test was performed in 28-day-old breastfed mice, age-matched mice that had NEC as newborns, and MCC950-treated NEC counterparts (Fig. 5a). The results revealed that there was no obvious difference of swimming speed between groups at the acquisition phase (Fig. 5b), indicating the comparable physical strength between differently treated mice. However, longer escape latency in the spatial training test (Fig. 5b) and decreased crossing-platform times (Fig. 5c) in the probe trial were observed in mice subjected to NEC compared with the breastfed controls. Indeed, all the impaired performance in Morris water maze test caused by NEC exposure was greatly counteracted by MCC950 treatment (Fig. 5 b and c). Intriguingly, NEC exposure in early life could result in significantly decreased expression of MBP, a structural and signaling molecule keeping the integrity of the myelin sheath as well as neuron normal functions enabling rapid and efficient signal propagation, even at 33 days postnatal (p33) as assessed by western blot. Paralleled with MBP reduction, the expression of NeuN, an important neuron marker, was also obviously decreased in NEC group compared to the control, indicating neuronal loss upon NEC induction, whereas the myelin and neuronal loss were largely rectified in mice that had been treated with MCC950 early in life (Fig. 5d).

## Discussion

Current treatment available for NEC, including feed cessation, parenteral nutrition, administration of broad-spectrum antibiotics, and surgical intervention [17], shows unfavorable therapeutic effect and poor prognosis. Parenteral nutrition usually increases the risk of metabolic abnormalities and liver injury, while prolonged exposure to antibiotics will lead to microbiota imbalance, drug resistance, and secondary infection [18, 19]. Although surgical intervention in NEC could control enteric content spillage and remove necrotic intestine, severe complications like intestinal stenosis and short bowel syndrome commonly develop [20]. More importantly, neurodevelopmental delay occurs in a considerable proportion of NEC survivors, especially in infants that underwent surgical intervention [21]; meanwhile, no targeted treatment for neurological impairment has been recommended currently. In this regard, better understanding of the pathogenesis of NEC and NEC-related brain injury as well as exploring additional effective therapeutic strategies are urgently needed.

Multiple pro-inflammatory cytokines play key roles in intestinal pathological damage of NEC. As the early inflammatory mediator, IL-1β is mainly secreted by activated macrophages, and its concentration significantly increased in the serum of NEC patients [22]. In our NEC mouse model, mature IL-1β levels of the intestine were also obviously upregulated, accompanied by increased expression of NLRP3, thus highlighting the implication of NLRP3 inflammasome activation in NEC. Excessive NLRP3 inflammasome activation and IL-1β production is capable of disrupting the intestinal mucosal barrier, directing neutrophils to injured site and promoting macrophage activation, representing promising therapeutic targets for NEC intestinal injury [23]. In the present study, we showed that MCC950, a specific small molecule inhibitor of NLRP3, significantly decreased the overall mortality of NEC mouse model, reduced pro-inflammatory cytokine expression (mature IL-1β, IL-6, and TNF-α), and greatly ameliorated the severity of histological damage in the intestine of NEC mice. Therefore, our studies indicated that NLRP3 inflammasome activation should be a pivotal mechanism of NEC development; meanwhile, NLRP3 inhibition could offer protective effects on NEC intestinal damage.

Compared to the extensive studies of intestinal injury, mechanisms of NEC-induced acute brain inflammation and long-term cognitive dysfunction remain far from elucidation; here for the first time we proved that NLRP3 inflammasome activation also participated in the pathogenesis of NEC-associated brain injury, as evidenced by the increased expression of NLRP3 in hippocampus and cerebral cortex regions as well as elevated cleaved caspase-1 and mature IL-1β levels in brain tissue of NEC animals. IL-1β has been reported to mediate pro-inflammatory cytokine production, activate microglia, and disrupt the blood-brain barrier (BBB), thus is involved in a variety of neuroinflammatory diseases [24]. Pharmacological inhibition of NLRP3 inflammasome activation and IL-1β production with specific antagonists, such as MCC950, has exhibited plausible effects on preventing neurological impairment of multiple sclerosis, neurodegenerative diseases, and traumatic brain injury [25–27]. In line with these reports, our data revealed that administration of MCC950 dramatically reduced NLRP3, IL-1β, IL-6, and TNF-α expression in the brain of NEC mice, accompanied by the decrease of activated microglia and astrocytes, thus resulting in profound improvement of acute brain damage in NEC. Furthermore, the loss of myelin, characterized by decreased expression of MBP, is closely related to neuron dysfunction and cognitive impairment, and demyelination has been found to be a key feature in the pathogenesis of a series of neurological disorders such as stroke, Alzheimer’s disease (AD), and Huntington’s disease [28, 29]. Our current data showed that MBP together with NeuN levels were significantly downregulated even at 3 weeks after NEC induction, while administration of MCC950 in early life of NEC pups efficiently restored brain MBP and NeuN expression. Consequently, MCC950-treated mice presented much better performance in MWM test than NEC controls, suggesting the reverse of neurodevelopmental delay caused by NEC. These findings together with the fact that MCC950 could pass through the blood-brain barrier [30] make it a promising drug candidate for intervention of brain injury in NEC.

Variety of exogenous and endogenous stimuli, including pathogen-associated molecular patterns (PAMPs) and damage-associated molecular patterns (DAMPs), can activate NLRP3 inflammasome. However, the triggers of NLRP3 inflammasome activation, especially in brain tissue, remain elusive under NEC conditions. Accumulating evidence has proved that neurodevelopmental delay occurs in a large proportion of NEC survivors, characterized by more severe neurocognitive injury than prematurity alone, thus suggesting a link between intestinal injury and brain damage [31]. Here, we found a positive correlation between the increment of IL-1β/IL-18 in the intestine and that in the brain, indicating that NLRP3 inflammasome activation in the brain of NEC mice correlates with the inflamed intestine. Since intestinal track holds a huge amount of microbes, thus, the microbial components and metabolites as well as necrotic tissue-released factors may comprise the major source of NLRP3 inflammasome activator in intestine of NEC individuals. Additionally, increasing studies [32, 33] have discussed the impact of gut microbiota alteration and intestinal damage on brain function, which could be mediated by enterogenic neurotransmitters, microbial metabolites, and inflammation-associated mediators (cytokines, et al.). For instance, the peripheral accumulation of phenylalanine and isoleucine, which was caused by the alteration of gut microbiota composition, has been reported to contribute to AD-associated neuroinflammation by stimulating the differentiation of pro-inflammatory T helper 1 (Th1) cells and activating microglia [34]. A recent study has demonstrated that intestinal damage-induced secretion of endogenous TLR4 ligands-high-mobility group box 1 (HMGB1) in the intestine could facilitate microglial cell activation in the brain and correlate with neurological impairment of NEC [35]. Considering the association of NLRP3 inflammasome activation in the intestine and in the brain tissue of NEC mouse model, gut-derived mediators under NEC scenario may do contribute to brain NLRP3 inflammasome activation, and additional studies need to be conducted to identify the exact initiators and detailed mechanisms.

## Conclusions

Collectively, our current study discloses that NLRP3 inflammasome activation may be closely related to intestinal and brain injury of NEC. Specific inhibition of NLRP3 with MCC950 exerts potent protective effects on NEC and NEC-induced acute brain damage as well as long-term cognitive impairment, therefore opening up a new therapeutic approach for infants suffering from NEC.

## Supplementary Information

**Additional file 1.** Supplementary Data.

## Data Availability

All data generated or analyzed during this study are included in this published article and its supplementary information files.

## Abbreviations

NEC: Neonatal necrotizing enterocolitis
NLRP3: Nucleotide-binding and oligomerization domain (NOD)-like receptor protein 3
IL-1β: Interleukin-1β
IL-6: Interleukin-6
TNF-α: Tumor necrosis factor-α
MBP: Myelin basic protein
MWM: Morris water maze
BBB: Blood-brain barrier
AD: Alzheimer’s disease
NO: Nitric oxide
ROS: Radical oxidative stress
OPCs: Oligodendrocyte precursor cells
PAMPs: Pathogen-associated molecular patterns
DAMPs: Damage-associated molecular patterns
Th1: T helper 1
HMGB1: High mobility group box 1
