# Supplementary material for: Blockage of NLRP3 inflammasome activation ameliorates acute inflammatory injury and long-term cognitive impairment induced by necrotizing enterocolitis in mice
Source: J Neuroinflammation. 2021 Mar 6;18:66. doi: 10.1186/s12974-021-02111-4 (PMC7937302; doi:10.1186/s12974-021-02111-4)
Supplement: Supplementary file 1 — Additional file 1. Supplementary Data. [file 12974_2021_2111_MOESM1_ESM.pdf]

Supplementary Data

Blockage of NLRP3 inflammasome activation ameliorates acute inflammatory injury and long-term cognitive impairment induced by necrotizing enterocolitis in mice

Fangxinxing Zhu, Lingyu Wang, Zizhen Gong, Yanyan Wang, Yanhong Gao, Wei Cai , Jin Wu

* **Correspondence**: Jin Wu, wujin@xinhuamed.com.cn; or Wei Cai, caiw204@sjtu.edu.cn

Inventory of Supplementary Data

Supplementary Figures and Legends

- Supplementary Figure 1. mRNA expression levels of NLRP3, NLRP1, NLRC4 and AIM2 in intestine of breastfed control and NEC mice, related to Figure 1
- Supplementary Figure 2. NLRP3 inflammasome activation in brain of NEC mice correlates with the inflamed intestine, related to Figure 1 and 2
- Supplementary Figure 3. Intestinal and brain histopathological analysis of mice in control, NEC, NEC+NS and NEC+MCC950 group, related to Figure 3 and 4
- Supplementary Figure 4. The effect of MCC950 treatment on brain weights and brain/body weight ratios of NEC pups, related to Figure 4

- Supplementary Figure 5. MCC950 treatment could decrease neuroinflammation in cerebral cortex of NEC mice, related to Figure 4.

Supplementary Figures and Legends

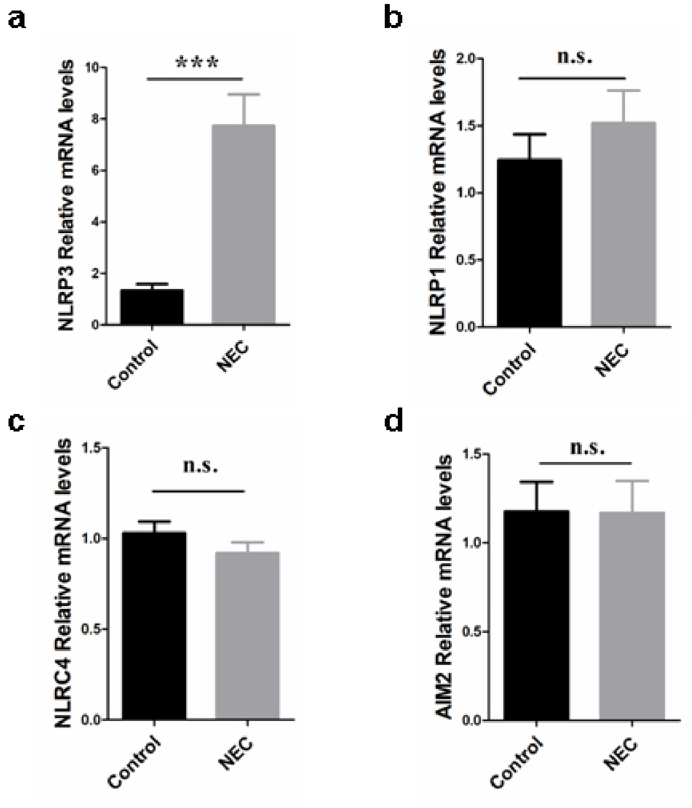

Supplementary Fig. 1 mRNA expression levels of NLRP3, NLRP1, NLRC4 and AIM2 in intestine of breastfed control and NEC mice. Real-time PCR analysis of NLRP3 (**a**), NLRP1 (**b**), NLRC4 (**c**) and AIM2 (**d**) levels in intestinal homogenates of control and NEC mice. ***: $p < 0.001$. n.s.: no statistically significant difference ($p > 0.05$). Error bars indicate s.e.m.

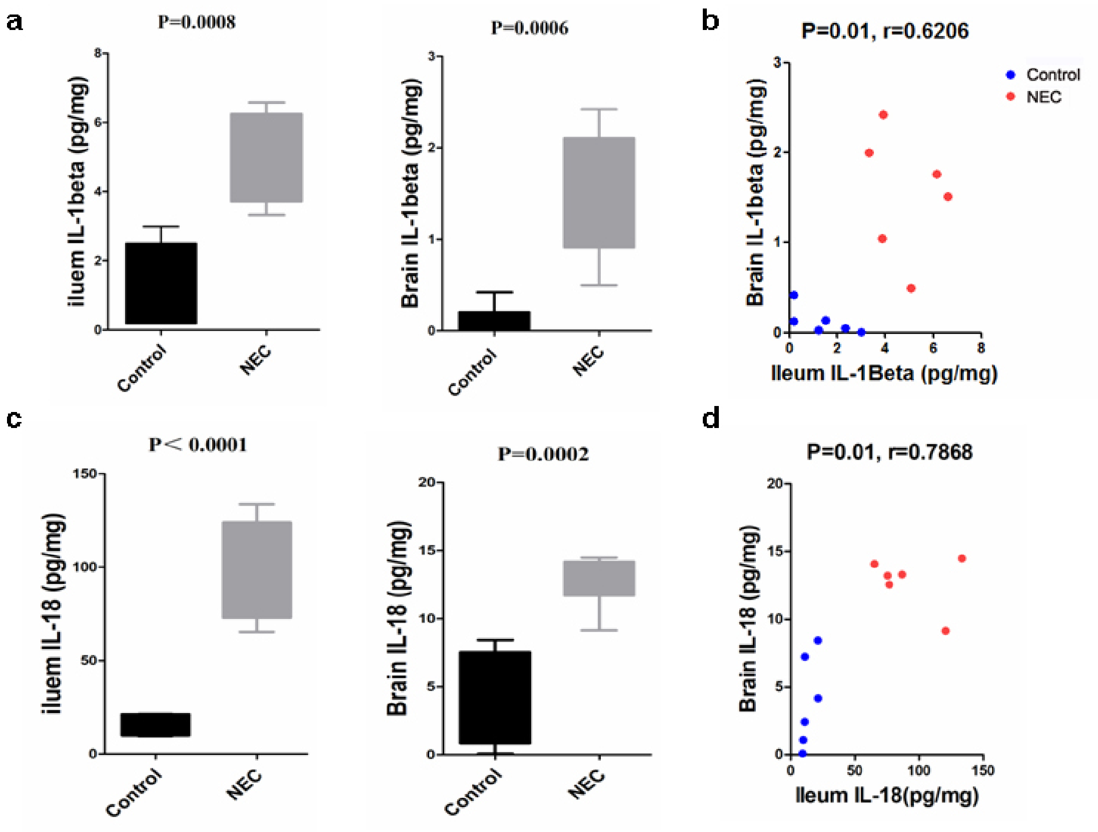

Supplementary Fig. 2 NLRP3 inflammasome activation in brain of NEC mice correlates with the inflamed intestine. **a** IL-1β levels in ileum and brain tissue homogenates of control and NEC mice were determined by ELISA (n=6/each group). **b** Pearson correlation of IL-1β levels of brain and ileum in control and NEC mice. **c** IL-18 levels in ileum and brain tissue homogenates of control and NEC mice were determined by ELISA (n=6/each group). **d** Pearson correlation of IL-18 levels of brain and ileum in control and NEC mice.

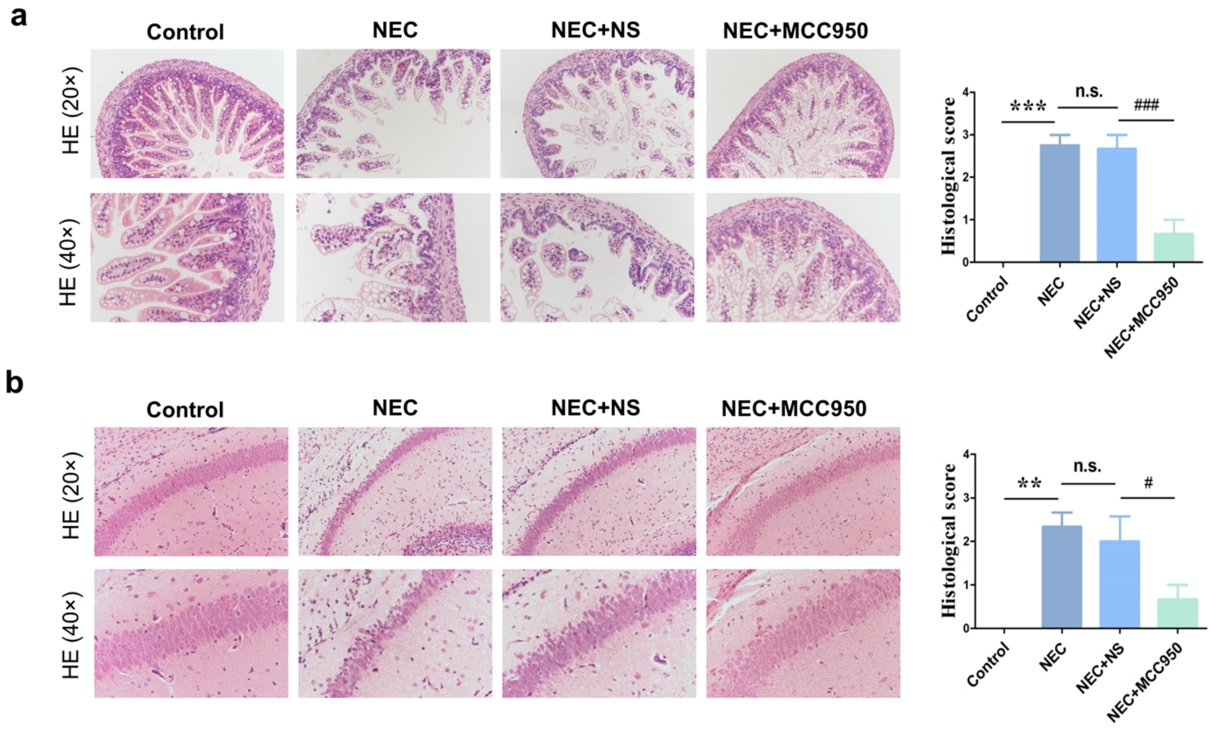

Supplementary Fig. 3 Intestinal and brain histopathological analysis of mice in control, NEC, NEC+NS (saline) and NEC+MCC950 group. **a** Representative H&E staining and histological score of intestinal sections from differently-treated mice. **b** Representative H&E staining and histological score of brain sections from differently-treated mice. **: $p < 0.01$, ***: $p < 0.001$ vs. control group; #: $p < 0.05$, ###: $p < 0.001$ vs. NEC+MCC950 group. n.s.: no statistically significant difference ($p > 0.05$). Error bars indicate s.e.m.

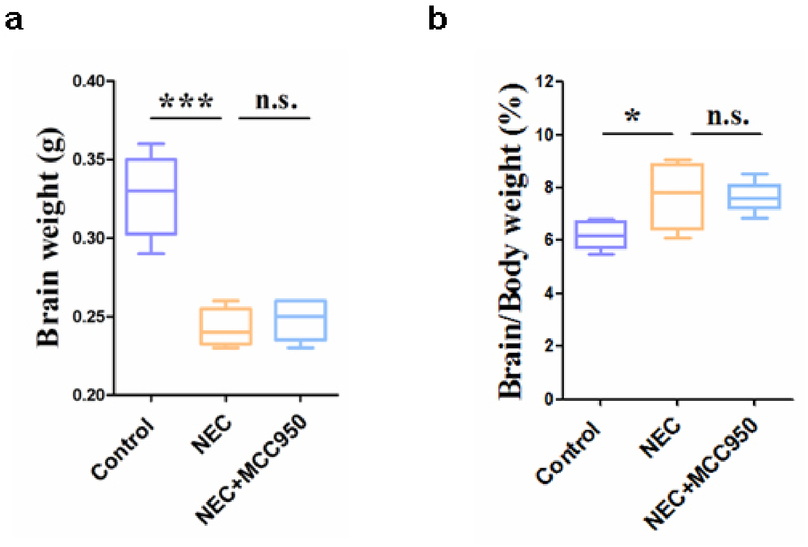

Supplementary Fig. 4 The effect of MCC950 treatment on brain weights and brain/body weight ratios of NEC pups. **a** Brain weights and **b** brain/body weight ratios of mice in control, NEC and NEC+MCC950 group. Brains in all groups were weighed immediately after harvest. Body weights were measured prior to brain harvest. *: $p < 0.05$, ***: $p < 0.001$ vs. control group; n.s.: no statistically significant difference ($p > 0.05$). Error bars indicate s.e.m.

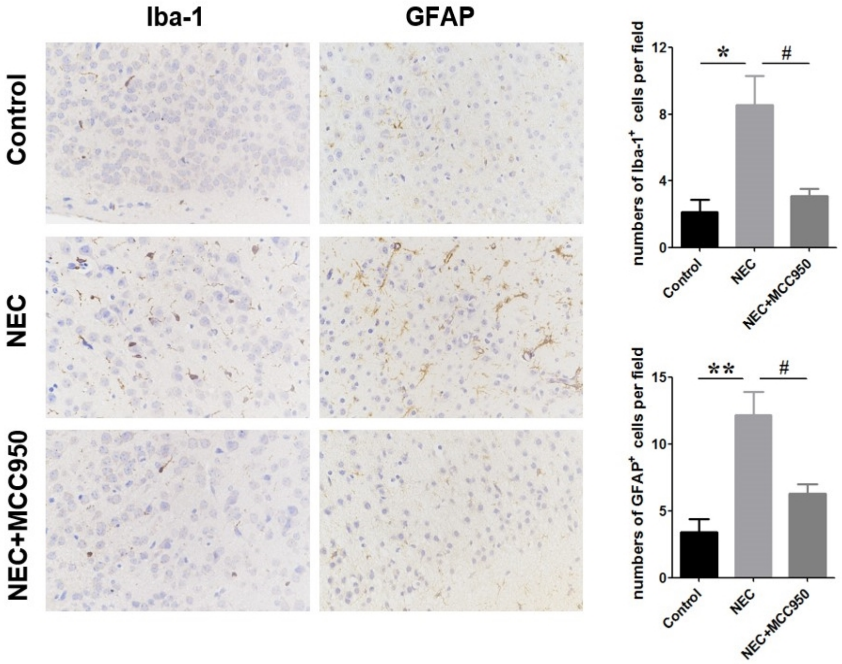

Supplementary Fig. 5 MCC950 treatment could decrease neuroinflammation in cerebral cortex of NEC mice. Representative Iba-1 and GFAP staining in the cerebral cortex of control, NEC and NEC+MCC950 mice. The numbers of Iba-1 or GFAP positive cells per field were quantified (3-6 fields/mice, n=3 mice per group).*: $p < 0.05$, **: $p < 0.01$ vs. control group; #: $p < 0.05$ vs. NEC+MCC950 group. Error bars indicate s.e.m.
